# Relationship between the positions of cytoplasmic granulation and the oocytes developmental potential in human

**DOI:** 10.1038/s41598-019-43757-8

**Published:** 2019-05-10

**Authors:** Xiao-fang Yi, Hong-Lin Xi, Si-Lin Zhang, Jing Yang

**Affiliations:** 1 Department of Reproductive Medical Center, Renmin Hospital of Wuhan University, Wuhan, 430060 China; 2 Department of Reproductive Medical Center, Affiliated Renhe Hospital of China Three Gorges University, Yichang, 443002 China

**Keywords:** Pluripotency, Pluripotency, Embryology, Embryology

## Abstract

To evaluate the relationship between the positions of cytoplasmic granulation and the oocytes developmental potential in human, we detected the developmental potentials of oocytes with centrally located cytoplasmic granulation (CLCG). The patients’ age, body mass index (BMI), Infertility duration, follicle stimulation hormone (FSH) levels, average stimulate ovulation days, gonadotropin (GN) total dose, fertilization rate, cleavage rate, high quality embryo rate, embryo utilization rate and pregnancy rate were analyzed. The results showed that there were no significant difference on patients’ age, BMI, infertility duration, FSH levels, average stimulate ovulation days, GN total dose, pregnancy rate and birth rate between CLCG group and control group in patients with BMI < 24 (*P* > 0.05). However, there was no significant difference in fertilization rate, cleavage rate, and high quality embryo rate in patients with BMI < 24 (*P* > 0.05). The pregnancy rate was low in both groups, but 35 and 15 healthy fetuses were born in each group. We also found that the central granulated area size did not affect fertilization rate, cleavage rate, embryo utilization rate, and high quality embryo rate (*P* > 0.05). These results suggested CLCG might be a normal morphology of oocyte. The oocytes from patients with or without CLCG had no significant difference in their developmental potentials. The patients who transferred CLCG embryos had successful delivery. The developmental potentials of oocytes with different CLCG grades had no obvious differences.

## Introduction

It is important to choose the high quality oocytes for *in vitro* fertilization (IVF) or intracytoplasmic sperm injection (ICSI) in assisted reproduction. With the opening of the one-child family planning policies in China, patients with advanced maternal age appealed the assisted reproductive technology (ART) to improve their fertility. Therefore, identification of easier predictors of the oocyte quality will be efficient to select embryos with high quality and treat infertility. In the past, most predictors are dependent on the morphological classification of the follicle, cumulus oocyte complex (COC), polar body, meiotic spindle and *et al*. At present, some intrinsic markers (mitochondrial status, glucose-6-phosphate dehydrogenase l activity) and extrinsic markers (apoptosis of follicular cells, levels of the transforming growth factor-β superfamily in follicular fluid or serum) have been used as good indicators of the oocyte quality.

In general, the cytoplasm of normal oocytes should exhibit uniform fine granules due to the presence of various organelles in the cytoplasm. However, granularity or granulation at the center of the cytoplasm is considered abnormal^1^. Serhal *et al*. firstly defined centrally located cytoplasmic granulation (CLCG): the central region of the oocyte cytoplasm appeared to be denser than other regions, forming a clear separation, and considered to have an adverse effect on embryonic development^1^. The mechanism of its occurrence is still unclear. Fancsovits *et al*.^2^ considered the occurrence of CLCG might be related to patient’s age and GN stimulation. The use of a large amount of GNs disrupts the developmental regulation and synchronization of oocytes, leading to problems in nuclear maturation and cytoplasmic maturation. Early studies^3^ confirmed that the complete polar body was the best nuclear maturation performance, while the broken polar body was an inconsistent nuclear maturation. Merviel *et al*. found that some patients had central granules in some oocytes, among which patients with high ratios (>75%) of CLCG compared to lower proportions showed a reduction in cleavage rate, pregnancy rate, and birth rate^4^. Patients exposed to the pesticide environment were found to have an increased incidence of central granulation of oocytes. Sun *et al*. believed that the use of GN during ovarian hyper stimulation (COH) in ART destroyed the developmental regulation and synchronization of oocytes, namely, the nuclear and cytoplasm maturity were not synchronized. Abnormal cytoplasmic granules may be a manifestation of cytoplasmic immature, and may also be associated with chromosomal abnormalities, leading to CLCG. Blastocysts formed from central granulating oocytes were performed biopsy aneuploidy up to 52.2%^5^. The CLCG oocytes had a negative impact on embryonic development^6^, especially in fertility rate^7^. However, another studies showed that there was no correlation between them^8,9^. Esfandiari *et al*. studied more than 500 CLCG oocytes and found that they had the same fertilization rate, embryonic development potential and pregnancy outcome as patients with normal cytoplasm^9^. Different conclusions may be related to the impact of multiple factors on patient outcomes. In view of this problem, our study adopted a progressive approach and analyzed it from three aspects. Firstly, to verify whether once patients oocyte with CLCG, all eggs affected the pregnancy outcome, we conducted analysis on the ICSI cycles of patients with body mass index (BMI) below 24 (BMI < 24), comparing the pregnancy outcome between CLCG group and control group. Secondly, the difference between oocytes with CLCG and with normal, from CLCG group with patients’ BMI under 24 (BMI ≥ 24), were also analyzed, so that we can verify whether the development potential of oocytes with CLCG of the same patient in the same cycle was lower than that of oocytes with normal morphology. At last, we tried to evaluate the central granulation area ratio by cell index extraction software to explore the effect of central granulation on embryonic development potential and birth outcome.

## Materials and Methods

### Statement

All experiments in this study were approved by the Ethic Committee of Life Science of Renmin Hospital of Wuhan University. All experiments were performed in accordance with relevant guidelines and regulations of the Ethic Committee of Life Science of Renmin Hospital of Wuhan University. We received agreement, permission, informed and signed consent from all patients included in the present study.

### Criteria for choosing patients and clinical data

A retrospective analysis of 331 patients, undergoing intracytoplasmic sperm injection (ICSI) from January 2010 to December 2018, in Reproductive Medical Center, Renhe Hospital Affiliated to Three Gorges University, for comparability, found only 255 patients with BMI < 24. Among these patients, 88 patients with some oocytes with CLCG were considered as the experimental group and 167 patients without CLCG oocytes as the control group. The reason of infertility mainly included: severe oligospermia or obstructive azoospermia in men, as well as conventional *in vitro fertilization* failure in patients.

### Controlled ovarian hyperstimulation proposal

According to the patient’s age, antral follicle number and basal endocrine selection, the controlled ovarian hyperstimulation proposals mainly included the long-term and short-term gonadotropin-releasing hormone agonist (GnRHa) protocols. Long-term GnRHa protocol was as follows: GnRHa was given from the luteal phase of the previous menstrual cycle, with a down-regulation period of 14–35 days. Up to the down-regulation criteria: endometrial thickness <5 mm, ovarian follicle diameter <5 mm, E2 < 50 pg/mL, FSH < 5 mU/mL, LH < 5 mU/mL, gonadotropin (Gn) was used to induce ovulation. When 1–2 dominant follicles had average diameters of more than 18 mm, it was considered that the dominant follicle had reached the maturity stage, HCG (10,000 U) was injected, and 34–38 h later, oocytes were taken by puncture. For short-term protocol, GnRHa (0.03–0.1 mg) was subcutaneously injected once daily on the second day of menstruation, and on the day 2–3, GN was injected to induce ovulation.

### Oocyte morphology assessment and cleavage stage embryo evaluation

When the oocyte was subjected to ICSI, it was observed under an inverted microscope (Leica, Germany, ×200) equipped with a constant temperature hot stage. The density of the central region of the oocyte was observed. The other regions were rough and formed a clear separation, which was called oocyte central granulation (CLGC). The CLGC oocytes classified into the experimental group, and the oocytes with normal morphology served as the control group. Prokaryotic evaluation was performed 16–18 h (D1) after ICSI. Two clear pronuclei were observed in normal fertilization, and the morphology of the embryos was evaluated in 42–44 h (D2) and 66–68 h (D3), and the number of embryo blastomeres, uniformity, and fragment ratio and multinuclear were recorded.

According to the morphological characteristics, the cleavage stage embryos were divided into 3 grades^10^. Grade I and II embryos were classed for high quality, and embryos at grades I-III were available for transplantation or freezing. Vitrification freeze-thaw technology (JY kit, Canada) was used for embryo cryopreservation. Two weeks after transplantation, blood β-HCG was determined. If it was elevated, ultrasound examination was performed 4–5 weeks after transplantation. The clinical pregnancy was confirmed after the intrauterine pregnancy sac was seen.

### Human oocyte CLGC grade

**Figure 1:**
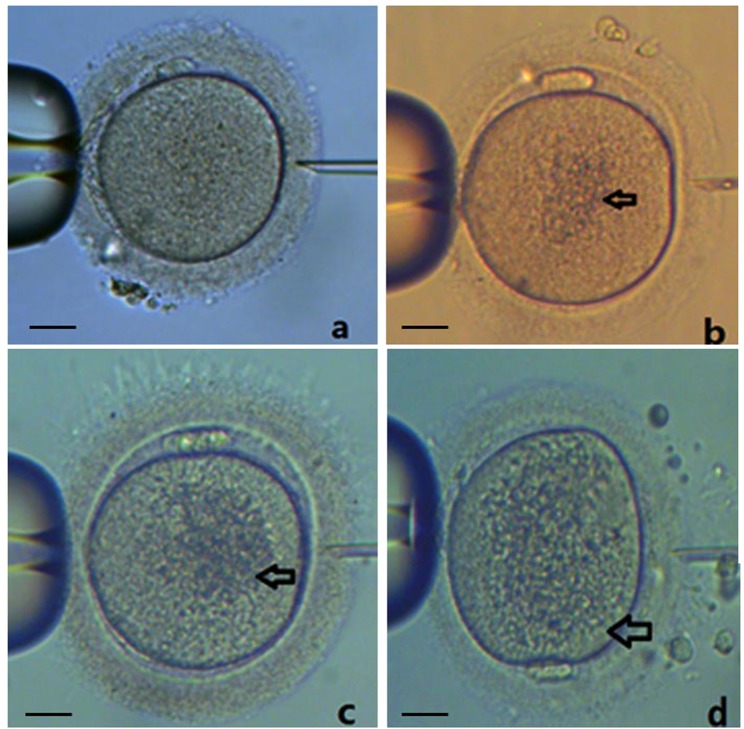
CLGC grading of human oocytes. The central granulated area size (S_center_) and oocyte area (S_oocyte_) of the equatorial plane were intercepted and the S_center_/S_oocyte_ ratio was calculated. (**a**) normal oocyte; (**b**) grade I: ratio of ≤1/4; (**c**) grade II: ratio of 1/4–1/2; (**d**) grade III: ratio of ≥1/2. Bar = 20 µM.

The images of CLGC oocytes were obtained. By cell index extraction software V1.0, the size of central granulated area (S_center_) and oocyte area (S_oocyte_) of the equatorial plane were intercepted, and the S_center_/S_oocyte_ ratio was calculated. The ratio ≤1/4 was recorded as grade I, 1/4–1/2 as grade II, and ≥1/2 as grade III (Fig. 1).

### Statistical analysis

Data analysis was performed using SPSS 17.0 software. The measurement data were analyzed by *t* test and the count data by *χ*^2^ test. *P* < 0.05 was considered statistically significant.

## Results

### Comparison of clinical data between two groups of patients

It was found that BMI and age might affect the results during the analysis of the clinical data, so the periodic patients were divided by BMI: BMI < 24 (Table 1) and BMI ≥ 24 (Table 2). However, we found that age was still the interference term in BMI ≥ 24, and the sample size was small (Table 2). Therefore, this study only analyzed patients with BMI < 24. There were no significant difference in patients’ age, BMI, Infertility duration, FSH levels, average stimulate ovulation days and GN total dose between the experimental and control group (*P* > 0.05).

**Table 1:** Clinical data of patients (BMI < 24) with CLCG oocytes and normal oocytes.

Groups	cycles (n)	age (years)	BMI	Infertility duration (years)	Baseline FSH level (mU/mL)	GN days (day)	GN dose (IU)
Normal	167	30.9 ± 4.99	20.45 ± 1.86	5.18 ± 4.14	7.17 ± 2.34	11.96 ± 3.86	2847.75 ± 1318.40
CLCG	88	29.94 ± 3.82	20.21 ± 1.84	4.26 ± 3.19	6.88 ± 1.87	11.66 ± 2.55	2776.99 ± 917.82
*P* value		0.088	0.333	0.070	0.320	0.613	0.654

**Table 2:** Clinical data of patients (BMI ≥ 24) with CLCG oocytes and normal oocytes.

Groups	cycles (n)	age (years)	BMI	Infertility duration (years)	Baseline FSH level (mU/mL)	GN days (day)	GN dose (IU)
Normal	57	32.51 ± 5.76	26.63 ± 2.68	5.61 ± 3.75	7.18 ± 2.73	12.67 ± 4.12	3249.12 ± 1288.98
CLCG	19	28.32 ± 5.19	25.75 ± 2.0	4.01 ± 3.44	6.62 ± 1.78	11.95 ± 2.34	2905.26 ± 1135.19
*P* value		0.006*	0.138	0.103	0.317	0.352	0.304

### Effect of CLCG on Pregnancy outcomes

The pregnancy rate was higher (38.98% v.s. 36.29%) and delivery rate was lower (25.42% v.s. 28.23%) in patients with CLCG compared with those without CLCG, however, there was no significant difference (*P* > 0.05) (Table 3).

**Table 3:** Pregnancy outcomes of patients (BMI < 24) with CLCG oocytes and normal oocytes.

Groups	cycles (n)	Clinical pregnancy rate (*n*, %)	Live birth rate (*n*, %)	In pregnancy (n)	Abortion (n)	Ectopic pregnancy (n)
Normal (1)	167	45 (36.29%)	35 (28.23%)	2	5	3
CLCG (2)	88	23 (38.98%)	15 (25.42%)	4	2	0
*P* value		0.725	0.691			

### Effect of CLCG on embryonic development potential

There was no significant difference between the experimental and control group in fertilization rate (62.2% *vs*. 62.5%), cleavage rate ((92.2% *vs*. 90.4%), high quality embryo rate (51.9% *vs*. 48.6%) and embryo utilization rate (72.6% *vs*. 68.3%) (*P* > 0.05) (Table 4). Because the transplanted embryos must be selected from the same type of the oocytes with or without CLCG, the number of patients enrolled was small and no statistical analysis was performed. However, both groups had normal fetal delivery (Table 5).

**Table 4:** The effects of CLCG and normal oocytes on embryonic development potential.

Groups	Oocytes (*n*)	Normal fertilization rate (*n*, %)	Cleavage rate (*n*, %)	High quality embryo rate (*n*, %)	Embryo utilization rate (*n*, %)
A	251	157	142	69	97
		(62.5%)	(90.4%)	(48.6%)	(68.3%)
B	185	115	106	55	77
		(62.2%)	(92.2%)	(51.9%)	(72.6%)
*P* value		0.934	0.62	0.608	0.46

**Table 5:** The effects of CLCG and normal oocytes on the pregnancy outcomes.

Grades	Transplantation cycle (n)	pregnancy cycle (n)	Live birth (n)
Normal	16	3	2
CLCG	15	5	5

### Effect of S_center_/S_oocyte_ Ratio on developmental potential of embryo

In the experimental group, there were 79 oocytes of grade I, 86 of grade II, and 20 of grade III. From grade I to grade III, there was no significant difference among the grades in fertilization rate (59.5% vs.65.1% *vs*. 60%), high quality embryo rate (39.5% *vs*. 63% *vs*. 44.4%), and embryo utilization rate (65.1% *vs*. 77.8% *vs*. 77.8%) (*P* > 0.05). Although the cleavage rate (92.2% *vs*. 90.4% *vs*.75%) was different, the theoretical number of multiple cells was less than 5 (Table 6). As a result, the number of patients enrolled was small and no statistical analysis was performed. The patients with grades I, II oocytes had normal fetal delivery (Table 7).

**Table 6:** The effects of S_center_/S_oocyte_ ratio on the developmental potential of the embryo.

Grades	Oocytes (*n*)	Normal fertilization rate (*n*, %)	Cleavage rate (*n*, %)	High quality embryo rate (*n*, %)	Embryo utilization rate (*n*, %)
I	79	47 (59.5%)	43 (91.5%)	17 (39.5%)	28 (65.1%)
II	86	56 (65.1%)	54 (96.5%)	34 (63.0%)	42 (77.8%)
III	20	12 (60.0%)	9 (75%)	4 (44.4%)	7 (77.8%)
*P* value		0.742	0.042	0.065	0.357

**Table 7:** The effects of S_center_/S_oocyte_ ratio on the pregnancy outcomes.

Grades	Transplantation cycle (n)	pregnancy cycle (n)	Live birth (n)
I	4	1	2
II	6	2	1
III	2	1	0

## Discussion

Studies^4,6^ have found that central granulation of oocytes had a negative effect on development potential, but this study, through stratified analysis, at least indicated that there was no difference in clinical outcomes between patients with BMI < 24 (Table 3). In the comparative analysis of the study subjects, age and BMI were found to be the interfering factors. Therefore, stratified study was conducted on BMI, and it was found that patients with BMI < 24 were comparable, while patients with BMI ≥ 24 needed to expand the sample size for study (Tables 1, 2). This study focused on the pregnancy outcome of fresh transplantation cycle and found no difference in pregnancy rate and delivery rate. These indicated that the pregnancy outcome of patients with CLCG oocytes was not affected. This result is consistent with the study of Esfandiari N *et al*.^9^.

Considering that it was possible that the embryos transplanted by CLCG patients may not necessarily be from CLCG eggs, the CLCG patients were analyzed from the perspective of CLCG and normal oocytes (Table 4). The results showed that there was no difference in fertilization rate, cleavage rate, high quality embryo rate and available embryo rate between CLCG and normal phase oocytes, which was further consistent with Esfandiari N *et al*.^9^. In this study, the subjects were limited to patients with both normal and CLCG-shaped oocytes, and their BMI was under 24. Other confound factors were excluded, which indicated that under the same condition, CLCG-shaped oocytes had the same developmental potential as normal oocytes. When the corresponding clinical outcomes were tracked, as there were fewer patients with single embryo transplantation, embryos derived from oocytes of CLCG morphology were likely to be transplanted together with embryos derived from other morphological origins. Therefore, this study only extracted the pregnancy outcomes of embryos derived from the same source of transplanted embryos, that is, all the transplanted embryos were from CLCG or normal morphology. A small number of cases were reported, but both groups had normal fetal birth, further indicating that normal fetal delivery can be achieved even with the transfer of CLCG-derived embryos.

Regarding the severity of central granulation, some centers classified them into mildness and severity^11^. Due to the interference of subjective factors, the results were inconsistent. For the first time, this study used cell index extraction software to quantify the central granulated area. The ratio of S_center_/S_oocyte_ was quantified and classified into three grades: grade I, grade II, and grade III. It was found that for patients with BMI < 24, the size of the area where CLCG appeared was independent of its developmental potential, further demonstrating that CLCG might be only normal oocyte morphology.

In conclusion, this study suggests that for infertile women with BMI < 24, there is no significant effect of CLCG on embryonic development potential, from the aspect of patient-to-patient, to the aspect of CLCG-to-normal oocytes, and to the aspect of CLCG oocytes grads. CLCG may be a normal form of oocyte. Furthermore, this study used the cell index extraction software to quantify the CLCG oocytes. However, large samples are needed to further refine the grading standards. Our present study did not take into account the follicular fluid. It will be a good direction to further elucidate the effect of CLCG on pregnancy outcomes from a mechanistic perspective. There is no direct relationship between the composition of follicular fluid and egg granulation as reported in the current literature. We will take into consideration and add relevant indicators, such as some indicators related to egg quality, which will be tested in the following experiments.
